# Reversible Martensitic Phase Transition in Yttrium-Stabilized ZrO_2_ Nanopowders by Adsorption of Water

**DOI:** 10.3390/nano12030435

**Published:** 2022-01-27

**Authors:** Elmar B. Asgerov, Anatoly I. Beskrovnyy, Nelya V. Doroshkevich, Carmen Mita, Diana M. Mardare, Dan Chicea, Mihaela D. Lazar, Alisa A. Tatarinova, Sergiy I. Lyubchyk, Svitlana B. Lyubchyk, Andriy I. Lyubchyk, Alexander S. Doroshkevich

**Affiliations:** 1 Joint Institute for Nuclear Research, 141980 Dubna, Russia; elmar@jinr.ru (E.B.A.); beskr@nf.jinr.ru (A.I.B.); nelyavik@gmail.com (N.V.D.); s191983@stud.spmi.ru (A.A.T.); 2 National Nuclear Research Center CJSC, Baku AZ1073, Azerbaijan; 3 Donetsk National Vasyl Stus University, 21021 Vinnitsa, Ukraine; 4 Faculty of Physics, Alexandru Ioan Cuza University of Iasi, Bulevardul Carol I nr. 11, 700506 Iași, Romania; cmita@uaic.ro (C.M.); dianam@uaic.ro (D.M.M.); 5 Research Center for Complex Physical Systems, Faculty of Sciences, “Lucian Blaga” University of Sibiu, Dr. Ion Ratiu str. no. 5–7, 550012 Sibiu, Romania; dan.chicea@ulbsibiu.ro; 6 National Institute for Research and Development of Isotopic and Molecular Technologies, 400293 Cluj-Napoca, Romania; diana.lazar@itim-cj.ro; 7 REQUIMTE, Faculdade de Ciências e Tecnologia, Universidade Nova de Lisboa, Quina de Torre, 2829-516 Caparica, Portugal; se.lyubchyk@fct.unl.pt (S.I.L.); s.lyubchyk@fct.unl.pt (S.B.L.); 8 Research Centre in Industrial Engineering Management and Sustainability, Lusófona University, Campo Grande, 376, 1749-024 Lisboa, Portugal; p6193@ulusofona.pt; 9 Nanotechcenter LLC, Krzhizhanovsky str., 3, 03680 Kyiv, Ukraine; 10 Donetsk Institute for Physics and Engineering named after O.O. Galkin, 03028 Kyiv, Ukraine

**Keywords:** nanopowders, zirconium oxide nanoparticles, adsorption phase transition, polymorphism in zirconium dioxide, size effect of structural stabilization

## Abstract

The present study was aimed at revealing the influence of the mechanical stress induced by water molecule adsorption on the composition of crystalline phases in the ZrO_2_ + 3 mol% Y_2_O_3_-nanoparticles. Three basic methods were used to determine the phase transition: neutron diffraction, Raman microspectroscopic scanning, and X-ray diffraction. The fact of reversible phase-structural β → α transformation and the simultaneous presence of two polymorphic structural modifications (β is the phase of the tetragonal syngony and α of monoclinic syngony in nanosized particles (9 nm)) under normal physical conditions was established by these methods. An assumption was made regarding the connection of the physical mechanism of transformation of the extremely nonequilibrium surface of nanoparticles with electronic exchange of the material of the near-surface layer of nanoparticles with the adsorption layer through donor–acceptor interaction. The principal possibility of creating direct-acting hydroelectric converters based on nanoscale YSZ (Yttria-Stabilized Zirconia) systems due to the reversible character of the considered effect was shown.

## 1. Introduction

Due to the development of nanotechnology, the study of subtle structural effects associated with exposure of external physical factors to nanoscale objects has become relevant [1,2]. An extremely interesting object in this respect is nanostructured systems based on Zirconium oxide under hydration conditions. The adsorption of water on the surface of the nanoparticles leads to the destruction of ZrO_2_-ceramics as a result of the propagation of the β *→* α (tetragonal-monoclinic, T-M) phase transformation from the surface to bulk of the material [2]. This transformation has a martensitic character, i.e., it goes forward at the speed of sound and requires a certain amount of material for the accumulation of the required number of elastic stresses [3,4]. It is commonly assumed that in nanoparticles smaller than 30 nm, T-M transformations cannot be realized due to the lack of the necessary volume for accumulation of stresses and propagation of the phase transformation front [5,6]. However, the results of [7,8], in which the effect of the so-called “chemoelectronic conversion” was shown, indicates that the T-M transformation still can be able in nanoscale objects. Moreover, it is shown that in nanoparticles, this transition has a reversible character. The confirmation of this fact is an extremely urgent task for fundamental science as for applications. In particular, changing parameters of the crystal lattice are accompanied by charge exchange of the nanopowder system with the external environment, which is extremely interesting for practical applications (electronics, power engineering, and so on). However, the study of such adsorption transformation is a technically complex task.

The general problem of the study of nanostructured systems by diffraction methods (X-ray, neutronography) is the large incoherent scattering ability of nanosized crystals (form-factor) and the high level of surface mechanical stresses due to the presence of an adsorption hydrate shell covering the nanoparticle surface. As a result, diffraction patterns from nanostructured objects are broad, contain a large level of incoherent background, and have a low reflex intensity. The intensity of M-phase reflexes induced by water adsorption does not usually exceed the error level, which does not allow the unambiguous identification of the considered adsorption effect using a single method.

The research methods themselves also have an individual limitation. The limitations of the applicability of XRD (X-ray diffraction) methods are connected to a relatively small depth of X-ray penetration (limited to a surface layer of several microns, depending on the atomic weight of the atoms in the sample). Such a “surface” study often gives distorted information about the structure of bulk layers of a material, especially if there is a gradient of properties (a gradient in the size and chemical composition of ceramic grains, inhomogeneous hydration of compacts, etc.). Neutronography, due to the relatively high penetrating power of neutrons (due to the relatively small scattering cross-section) at the same resolution (0.01%), allows the collection of information from a significantly larger volume of material than in the case of XRD. However, the use of neutron diffraction in these studies is limited by the absorption of neutrons by water molecules. This problem is partially solved by the replacement of water to deuterium oxide. It should be noted that the diffraction pattern in X-ray diffractometry and neutronography can be obtained in different ways (by variation of the 2*θ* angle and the use of the TOF method, respectively). Consequently, weak reflexes recorded by several methods in the region of the corresponding interplane distances are very likely to indicate that they belong to the desired phase. Thus, the combined use of X-ray diffraction and neutronography methods will significantly increase the probability of detecting small effects in nanopowder objects.

In addition, an alternative to diffraction methods is a spectrometric method for conducting structural studies. In particular, the Raman light scattering method has proven itself in structural studies. Raman light scattering methods are sensitive to the short-range ordering of the arrangement of atoms in a structure and make it possible to investigate the subtle features of the real structure of crystals. The vibrations of complex anions in the crystal structure, the bonding forces inside of which considerably exceed the bonding forces with the cation sublattice, can be studied with their help. In this case, isomorphic substitutions in cationic positions affect the position of vibration bands of complex anions. Knowing the spatial group of the crystal and the group of positional symmetry of a complex ion in the structure, it is possible to calculate the number of vibration bands of this ion in the main spectral regions. These peculiarities of interaction with the substance of radiation (in the optical and X-ray ranges) and particles (neutrons), as well as various principles of obtaining and processing data, make it possible to obtain the set of data required for unambiguous identification of the phase composition of nanoscale objects.

The present work aims to determine a weakly expressed adsorption-induced reversible phase transformation in a YSZ-nanopowder system using individual features of the listed sensitive analytical methods.

## 2. Materials and Methods

**Objects of research and methods of obtaining them.** As the object of investigation, ZrO_2_ + 3 mol% Y_2_O_3_ nanopowders were used. The ZrO_2_ + 3 mol% Y_2_O_3_ nanopowders were synthesized by the co-precipitation technique using ZrOCl_2_·9H_2_O salt (JSC SVK, Dnipro, Ukraine) [9]. All used chemicals were of high chemical purity (SiO_2_ < 0.008 wt%, Fe_2_O_3_ < 0.01 wt%, Na_2_O < 0.01 wt%). The appropriate amounts of Y_2_O_3_ (Merck, Darmstadt, Germany) were dissolved in nitric acid. Thereafter, zirconium (0.75 mol/L) and yttrium (1 mol/L) salts were mixed via a propeller stirrer for 30 min and were subsequently added to an aqueous solution of the precipitant (25% NH_4_OH) with constant stirring. Sediments were mixed for 1 h at room temperature at pH > 9. Then sediments were repeatedly washed with distilled water and filtered on a suction filter. Washing of sediments was carried out until a negative test for Cl^−^ ions was obtained with the use of a silver nitrate solution. After washing and filtration, the hydrogel was dried in a microwave furnace with an output power of 700 W and at a frequency of 2.45 GHz. The calcination of dried zirconium hydroxides was carried out in resistive furnaces at 400 °C with a dwelling time of 2 h.

**Measurement technique.** Powder diffractograms (X-ray- and Neutron diffraction) were measured in humid (saturated in H_2_O or D_2_O) conditions at room temperature, and in a dry state at 200 °C. Raman studies of the samples were carried out at room temperature immediately after saturation or immediately after drying at 200 °C for 4 h. To implement humid conditions, the samples were saturated in a vapor of deuterium oxide atmosphere for neutronography or water vapor atmosphere for X-ray diffraction and Raman spectroscopy for 24 h. The samples adsorbed up to 10–15 wt% of the total mass during saturation. The moistened state of the sample was chosen as the initial one for technical reasons.

**Figure 1:**
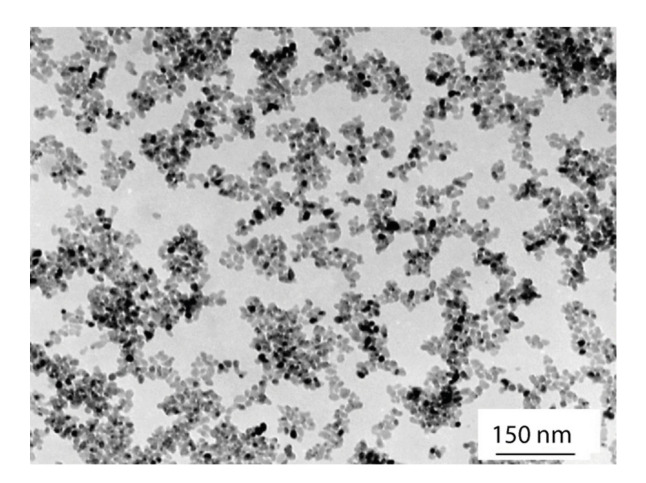
TEM image of a nanopowder with the composition ZrO_2_ + 3 mol% Y_2_O_3_, 400 °C, 2 h.

**The spatial structural organization** of the samples was studied by transmission electron microscopy (TEM) JEOL, JEM 200A, Tokyo, Japan) (Figure 1).

**X-ray diffraction procedure.** The crystal structure of the nanopowder samples in X-ray frequency range was studied using an Empyrean PANalytical X-ray diffractometer (Malvern Panalytical, Malvern, UK) with CuKα radiation emitted from the Ni filter. Reflection change profile was adjusted in the range of 30° < 2*θ* < 45° using Lorentzian-peak functions without imposing any structural constraint.

The ratio of the integrated peak intensities: $I_{111}^{m}$, $I_{-111}^{m}$, $I_{101}^{t}$ was used for the calculation of the XRD-derived monoclinic volume fraction *V^m^*. The molar fraction of the content of M-ZrO_2_ was calculated as:

(1) $$X^{m}=\frac{I_{111}^{m}+I_{-111}^{m}}{I_{111}^{m}+I_{-111}^{m}+I_{101}^{t}}$$

where $I_{111}^{m}$, $I_{-111}^{m}$ are the intensity of the peaks representing the crystal orientation (111) and (−111) of M-ZrO_2_, and $I_{101}^{t}$ is the intensity of T-ZrO_2_ with the crystal orientation of (101) [10].

The volumetric fractions of the M-ZrO_2_ and T-ZrO_2_ phases from the intensities of the diffraction peaks (111) and (−111) of monoclinic and line diffraction (101) tetragonal were calculated as:

(2) $$V^{}=\frac{1.31X^{}}{1+0.31X^{}}$$

For a proper application of this equation, a fitting procedure for the X-ray pattern was performed using a Lorentzian or Gaussian curve to determine peak intensity. In the ZrO_2_ tetragonal P4_2_/nmc structure, the six atoms were localized in the following special positions: 4d for oxygen, corresponding to a tetrahedral site centered on Zr(Y) atoms, and 2a for Zirconium.

**Neutron diffraction.** Neutron diffraction experiments were carried out on the diffractometer DN-2, located on a high-flux pulsed neutron reactor Fast Neutron Pulse Reactor IBR-2 (Laboratory of Neutron Physics named I.M. Frank, Joint Institute for Nuclear Research, Dubna, Russia) [11]. A powder weighing 2 g in an aluminum foil cylinder was a sample for neutronographic research. The diffraction patterns were measured at a scattering angle of 2*θ* = 174°. The measurement time of one diffraction pattern was 4 h. To record the diffraction patterns, the time-of-flight method was used; the total neutron flux on the sample was 5 × 10^6^ n/cm^2^∙s, the spatial resolution *Δd/d* at the interplanar distance at a scattering angle of 2*θ* = 174° was close to 0.01. Neutron diffraction data were processed by the Rietveld method using the FullProf program.

The energy spectrum of slow neutrons has a continuous (Maxwell) character [12]. The time of flight (TOF) method was used for the analysis of the slow neutron energy as a slow neutron source a pulsed nuclear reactor IBR-2 was used. The position of the diffraction peaks on the time scale was determined by the following condition:

(3) $$t=\frac{L}{v}=\frac{\lambda mL}{h}=\frac{2mld_{hkl}\sin\theta }{h}$$

where *L* is the total transit distance from the neutron source to the detector, *v* is the neutron velocity, *λ* is the neutron wavelength, *m* is the neutron mass, *h* is the Planck constant, *d_hkl_* is the interplanar distance, and *θ* is the Bragg angle.

The functional dependence of the resolution *R* on the interplanar distance *d* (or the transmitted pulse Q) [13] was calculated as:

(4) $$R=\frac{\Delta d}{d}=\sqrt{{\left(\frac{\Delta t_{0}}{t}\right)}^{2}+{\left(\frac{\Delta \theta }{tg\theta }\right)}^{2}+{\left(\frac{\Delta L}{L}\right)}^{2}}$$

where *Δt*_0_ is the neutron pulse width, *t* = 252,778 *Lλ* is the total time of flight (*μ*s), *L* is the flying distance from the source to the detector (*m*), λ is the neutron wavelength (Å), and *θ* is the Bragg angle.

**Raman Spectroscopy.** The Raman LabRAM HR Evolution Horiba (Kyoto, Japan) spectrometer at a wavelength of the emitting laser of 633 nm was used to determine the phase composition of Zirconium dioxide powders after drying and after adding of deuterium oxide. The spectra were recorded at room temperature in the region of 150–1200 cm^−1^. All measurements were performed in triplicate. Calculations were carried out using the computer program Origin 9.2 (OriginLab Corporation, Northampton, MA, USA).

The group theory predicts 18 phonon branches for tetragonal zirconium dioxide from which 6 are vibrational Raman active modes are given by:

(5) $$Г=A_{1g}+2B_{1g}+3E_{1g}$$

where *A_1g_*—a mode of the oxygen motions along the *z*-direction only; *B_1g_*—a mode including Zirconium and oxygen displacement in the z-direction; and *E_1g_*—a mode consisting of displacements of oxygen and zirconium in the (x, y) plane [14].

According to the group theory, there are 36 lattice vibrational modes for monoclinic ZrO_2_ including infrared, acoustic, and others [15]:

(6) $$Г=9A_{g}+9A_{u}+9B_{g}+9B_{u}$$

The doublet found for the monoclinic ZrO_2_ at 181 cm^–1^ and 190 cm^–1^, with the adjacent tetragonal peak at 147 cm^−1^, is commonly used for the quantitative determination of monoclinic phase:

(7) $$V^{m}=\sqrt{0.19-\frac{0.13}{X^{m}-1.01}-0.56}\;\mathrm{with}\;X^{m}=\frac{I_{181}^{m}+I_{190}^{m}}{I_{181}^{m}+I_{190}^{m}+I_{147}^{t}}$$

where $I_{181}^{m}$, $I_{190}^{m}$ and $I_{147}^{t}$ are the integrated intensity of the monoclinic 181 cm^−1^ and 190 cm^−1^ and the tetragonal 147 cm^−1^ peaks, respectively. A common procedure consists of plotting the monoclinic volume fraction *V^m^* of the wet specimens against the monoclinic/tetragonal intensity ratio *X^m^* measured from the Raman spectra [16].

## 3. Results

**Electron microscopy data.** A typical electron microscopic image of the studied powders is shown in Figure 1.

It can be seen that the particles of the order of 9 nm are uniform in size and relatively well separated.

**Figure 2:**
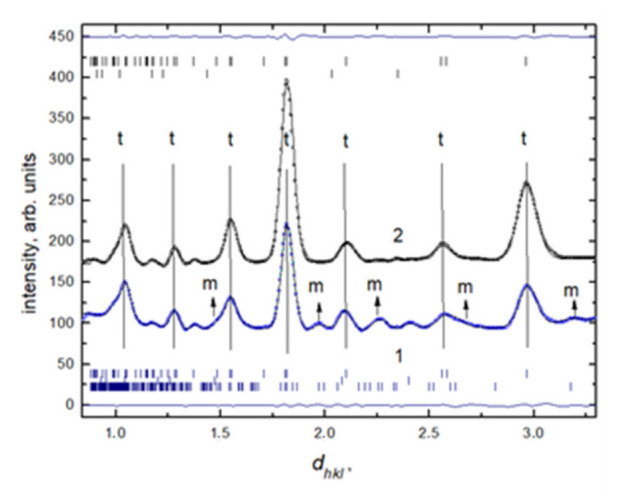
Neutron diffraction patterns of dry (black line, 2) and hydrated (blue line, 1) ZrO_2_ + 3 mol% Y_2_O_3_ nanopowder.

**Neutron diffraction data.** Neutron diffraction patterns of hydrated and dry ZrO_2_ + 3 mol% Y_2_O_3_ are shown in Figure 2.

The analysis of the neutron data showed that the nanostructured system saturated with deuterated water under normal physical conditions was in the tetragonal crystalline symmetry system P4_2_/nmc. The presence of a small amount of the monoclinic phase with P21/c symmetry should be noted. The values of the parameters of the unit cell of the tetragonal and monoclinic phases calculated from the diffraction data are given in Table 1. It can be seen that the desorption of deuterium oxide leads to the disappearance of the reflexes d_hkl_ = 1.47 Å, d_hkl_ = 1.97 Å и d_hkl_ = 2.27 Å, d_hkl_ = 2.67 Å, d_hkl_ = 3.17 Å corresponding to the M-phase on the diffraction patterns.

Thus, a model experiment with heating shows that annealing at 200 °C leads to complete reduction of the M-phase (~8% M-phase) in YSZ nanopowders. This effect confirms the above considerations regarding the destabilization of the T-phase by adsorbates. Vertical lines indicate the calculated positions of the structural diffraction peaks. The most intense peaks are denoted by the symbols “M” and “T” (for the monoclinic and tetragonal phases, respectively). The parameters of the unit cells of M- and T-phases in the ZrO_2_ + 3 mol% Y_2_O_3_ nanopowders, obtained from neutron spectra, are given in Table 1.

**Figure 3:**
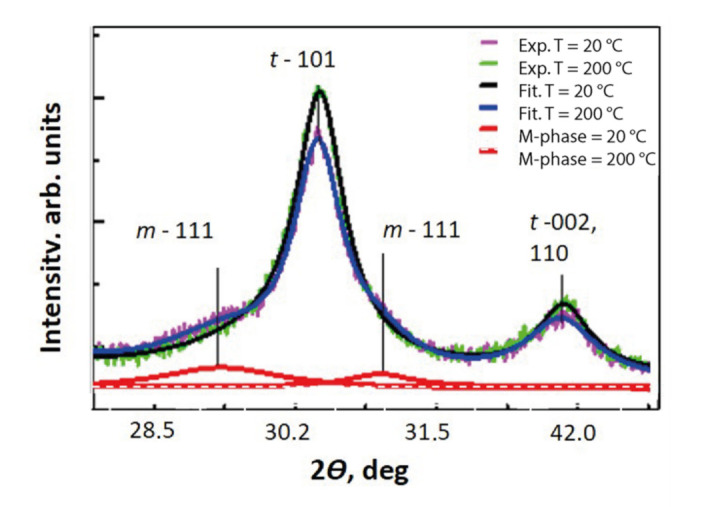
Experimental (green and purple lines) and calculated (green and black lines) XRD diffraction patterns, and mathematically selected M-phase signal (red lines) of ZrO_2_ + 3 mol% Y_2_O_3_ nanopowder sample, after saturation and drying at 200 °C, 30 min.

**X-ray data.** The XRD diffraction patterns of YSZ-nanopowders obtained under different conditions are shown in Figure 3.

The structure of the dry sample was identified as being tetragonal. The presence of the M-phase in samples saturated with H_2_O is very weak. The value of the M phase was found to be V_m_ ~ 3–4%, according to Equation (4), which indicates that the adsorption-induced phase transformation affects only a small part of the volume of the material. The parameters of the unit cells of the M- and T-phases in the ZrO_2_ + 3 mol% Y_2_O_3_ nanopowder with different degrees of moisture (H_2_O), obtained from the X-ray spectra, are given in Table 2.

**Figure 4:**
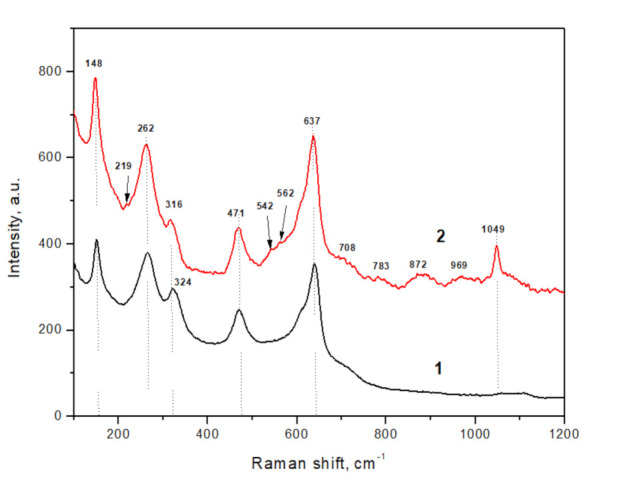
Raman spectra of dry (black line, 1) and deuterated (red line, 2) ZrO_2_ + 3 mol% Y_2_O_3_ nanopowders.

**Raman data.** Nanopowder of ZrO_2_ + 3 mol% Y_2_O_3_, dried at 200 °C, is characterized by a T-ZrO_2_ structural modification, according to Raman spectroscopy data, which is well expressed by intense peaks at 150, 265, 322, 469, and 639 cm^–1^ (Figure 4) [17,18].

Peaks of the monoclinic phase were absent in this sample. The ratio of the tetragonal peaks intensity I_265_/I_316_, which characterizes the ordering of the crystal lattice, was 1.3 in this case. The ratio of the I_262_/I_637_ peaks intensity, which characterizes the degree of tetragonality in the sample, was 1.1. This fact confirmed the presence of the tetragonal phase only.

The Raman spectrum of deuterated nanopowder corresponded to a mixture of tetragonal and monoclinic phases. The well-pronounced intense peaks detected at 148, 262, 316, 471, and 637 cm^−1^ correspond to the T-ZrO_2_ phase. A slight shift (2 cm^−1^) to the low-frequency region compared with the peaks of the dry powder indicated the presence of distortions in the crystal lattice, especially in Zr–O (Y–O) bonds. The low-intensity peaks at 219, 542, 562 cm^−1^, and a series of weakly intense peaks of H-group (708, 783, 835, 859, 872, 955, 1145, 1167 cm^−1^), the doublet found for the monoclinic ZrO_2_ at 181 cm^−1^ and 190 cm^–1^, and the adjacent tetragonal peak at 147 cm^−1^ were not observed in our case. However, the peak groups characteristic of the ZrO_2_ monoclinic phase and characteristic peak intensity ratios allow us to unambiguously establish its presence. The C-group (100–700 cm^−1^ region) corresponded to the M-ZrO_2_ phases [19,20,21]. The ratio of the intensities of the peaks of the tetragonal I_256_/I_316_ modification in this sample was 1.4, and the ratio of the intensities of the peaks I_262_/I_637_ was 0.9, which confirms the presence of two phases [22]. The estimated (Equation (6)) quantity of the M-ZrO_2_ phase was about ~5%. Thus, Raman spectroscopy confirmed neutron and X-ray diffraction data regarding the adsorption-induced formation of the monoclinic phase in ZrO_2_ + 3 mol% Y_2_O_3_ nanopowders.

## 4. Discussion

The experimental data obtained together indicate the realization of a reversible adsorption phase transformation in the nanopowder system under study.

The key issue of this discussion is the mechanism of this phase transformation and the structural element responsible for its implementation. The answer to these questions lies in understanding the mechanism of the high-temperature phase’s stabilization in YSZ materials.

There are many opinions regarding the mechanism of T-phase stabilization in the YSZ system. In [23,24,25], an explanation and experimental proof of the stabilization of ZrO_2_ in the cubic phase due to the nonstoichiometry of oxygen vacancies is given. The essence of such stabilization by nonstoichiometric defects is that, if the required concentration of anion vacancies is created in the material, then the local stresses arising, in this case, can keep the high-temperature cubic phase at room temperature. This explanation is also applicable to the stabilization of β-ZrO_2_ [2]. In this case, nonstoichiometry is the main factor determining the stability of high-temperature phases. Many researchers believe that the existence of high-temperature ZrO_2_ phases under normal conditions could be due to the stabilizing effect of impurities. Thus, in [26], enhancement of the formation and existence of high-temperature phases for the corresponding oxides through the interaction of Zr^4+^, Hf^4+^, Th^4+^, U^4+^ ions with water and hydroxyl ions was reported. This point of view is also expressed in [4,27]. A similar stabilizing effect is exerted by metal oxides dissolved in ZrO_2_, the presence of which in the lattice also leads to the appearance of oxygen vacancies [28]. It is believed that in this case, a stabilizing effect is exerted by the stresses created by oxygen vacancies, as in the case of nonstoichiometric stabilization. Thus, most of the known models describing stabilization point to the key role of the defective subsystem, namely oxygen vacancies in the stabilization of high-temperature phases in YSZ materials. Therefore, the changes in the subsystem of vacancy defects should lead to the destabilization of the high-temperature T-phase.

As can be seen from Table 2, the adsorption of water leads to a decrease in the volume of the crystal lattice cells of the T-phase by an amount of 0.127 A or 0.19%. It can be concluded that the adsorption of moisture leads to the densification of the surface layer of the material of nanoparticles concerning the volume, presumably as a result of the collapse of vacancies after localization of some electrons from the lattice to the energy levels of the adsorbate. Thus, the established effect, as one of the forms of recharging of oxide nanoparticles surface layer, confirms the adsorption mechanism of electron emission in ZrO_2_ + 3 mol% Y_2_O_3_ described in [7,8,29,30]. In the opinion of the authors, during adsorption processes, water molecules through the donor–acceptor interaction with impurity cations Y^3+^ lead to the localization of electrons from the local energy levels of surface atoms, thereby destroying the T-phase stabilizes bond between impurity ions Y^3+(−)^ and oxygen vacancies V^−(+)^ [4]. The subsequent increase in the internal energy of the crystal leads to the destabilization of the metastable T-phase and the formation of an equilibrium M-phase in an amount proportional to the probability of localization of electrons at the adsorption levels of adsorbates, described by the Fermi–Dirac statistics [2]. The recharging of ions probably leads to the creation of free charge carriers in the hydroelectric converters considered in [8,31].

The physical bases for the reverse transformation can be explained from the standpoint of thermodynamic principles [31,32,33]. Since the internal crystal energy *U* consists of the energy of the inner part of the crystal *U_v_* and the surface energy *σS* (*σ* is the specific surface energy; *S* is the surface area), *U* = *U_v_* + *σS* and *U_vα_*< *U_vβ_*< *U_vγ_*, and *σ_α_*> *σ_β_*> *σ_γ_*, then for *S* < *S_cr_* at room temperature, the high-temperature phase can be energetically favorable, i.e., the high-temperature phase is more stable for the smaller particle size. Thus, the reverse process (M-T transformation) is exclusively a size effect and cannot be realized in massive bodies precisely because of the negligible surface energy. Thus, in the case of nanoscale objects, there is the possibility of the direct electrical conversion of the energy of adsorption processes into an electrical form through the cyclic action of water vapor/deuterium oxide.

## 5. Conclusions

Using the Raman spectroscopy, neutron and X-ray diffraction methods, a unique T-M phase transformation in nanosized YSZ objects upon water adsorption was established. The M-ZrO_2_ phase appearing at a quantity about ~5% after moisture saturation from the gas atmosphere under normal physical conditions was shown.

A decrease in the volume of the crystal lattice cells of the T-phase by an amount of 0.19% was established, as a result of moisture adsorption by surfaces.

An assumption was made regarding the connection of the physical mechanism of transformation of the extremely nonequilibrium surface of nanoparticles with the collapse of vacancies in the near-surface layer due to the donor–acceptor interaction of the electron subsystem of nanoparticles with the adsorption layer (Y^3+(−)^–V^−(+)^–bond).

The possibility of the adsorption of hydroelectric conversion by transformational mechanism was shown by the example of ZrO_2_ + 3 mol% Y_2_O_3_ nanocrystals.

The above explanations of the effect are qualitative in nature. Additional research is required. The question of realizing the mechanism for the determined T-M transformation remains open. For a better understanding of this phenomenon, additional analysis of the process mechanism and its thermodynamics are necessary. The obtained results provide new perspectives on the fundamental and applied aspects of powder nanotechnologies.

## Figures and Tables

**Table 1:** Parameters of the unit cells of T- and M-phases in the ZrO_2_ + 3 mol% Y_2_O_3_ nanopowder according to neutron diffraction data.

	a, Å	b, Å	c, Å	α, Å	β, deg	γ, deg
T-phase	3.621(3)	3.621(3)	5.178(4)	90.00	90.00	90.00
M-phase	5.128(5)	5.246(0)	5.288(1)	90.00	100.16(3)	90.00

**Table 2:** Parameters of unit cells of T- and M-phases in the ZrO_2_ + 3 mol% Y_2_O_3_ nanopowder with different degrees of moisture.

	a, Å	b, Å	c, Å	α, Å	β, deg	γ, deg	Volume, Å^3^
T-phase (dry)	3.612(1)	3.612(1)	5.186(2)	90.00	90.00	90.00	67.653(9)
T-phase (wet)	3.610(4)	3.610(4)	5.182(2)	90.00	90.00	90.00	67.551(2)
M-phase (wet)	5.118(0)	5.241(2)	5.274(4)	90.00	100.18(4)	90.00	139.253(8)

## Data Availability

Not applicable.
